# Association Between Parental Smoking Status and Adolescent Mental Health: Population-Based Study

**DOI:** 10.2196/73588

**Published:** 2026-01-05

**Authors:** Soo Jin Kwon, Hyejin Kim, Ji-Su Kim, Bo Han Kim

**Affiliations:** 1 Department of Nursing, Ansan University, Ansan, Republic of Korea; 2 Department of Nursing, Chung-Ang University, 84, Heukseok-ro, Dongjak-gu, 06974, Republic of Korea, 82 28206686

**Keywords:** parental smoking, adolescent mental health, suicidal ideation, melancholy, cognitive stress

## Abstract

**Background:**

Adolescents’ mental health problems significantly affect their long-term psychological and physical health. Although peer influence grows during adolescence, parental influence remains critical. Parental smoking is associated with behavioral problems in adolescents.

**Objective:**

This study aimed to investigate the association between parental smoking, particularly maternal smoking, and adolescents’ mental health outcomes in South Korea, as research in this area is limited.

**Methods:**

We analyzed data from the nationwide Korea National Health and Nutrition Examination Survey from 2012 to 2017. A total of 2761 adolescents were included in the final analysis after excluding those with missing data. We used ANOVA and chi-square tests to compare adolescents’ and parents’ baseline characteristics and mental health. In addition, multiple logistic regression analyses were conducted to examine the association between parental smoking status and adolescents’ mental health.

**Results:**

Our logistic regression analyses revealed that mothers’ current smoking habits were significantly associated with their adolescents’ cognitive stress (odds ratio [OR] 1.65, 95% CI 1.06‐2.56), experiences of melancholy (OR 2.09, 95% CI 1.20‐3.65), and suicidal ideation (OR 2.39, 95% CI 1.17‐4.88). Furthermore, adolescents whose mothers were current smokers and had cognitive stress demonstrated higher cognitive stress (OR 2.09, 95% CI 1.12‐3.90), melancholy (OR 2.27, 95% CI 1.10‐4.71), and suicidal ideation (OR 2.74, 95% CI 1.21‐6.23) than those whose mothers were not smokers and had no cognitive stress.

**Conclusions:**

Efforts to improve adolescents’ mental health require considering their mothers’ smoking status and stress levels. This highlights the need to develop programs to enhance adolescent mental health, manage maternal stress, and promote smoking cessation.

## Introduction

Adolescence is a critical period for the development and maintenance of social and emotional habits that are important for mental health [1]. Mental health issues during adolescence have considerable long-term impacts on one’s physical and psychological status, with parental behaviors playing a critical role in this developmental stage [2], including in school connectedness [3,4], emotional and physical health [5], substance misuse [6], and suicide-related injury risk [7,8]. Therefore, prevention and early intervention are essential for effective mental health management and improved social outcomes [9].

Several variables affect the mental health of children and adolescents, including school-, peer-, and family-related factors [10]. However, there is renewed interest in family-related factors that affect children’s mental health. In particular, the relationship between parental and children’s mental health requires further verification and support [11].

Researchers have identified several parental health-risk behaviors as predictors of poor mental health in children. Parental smoking, particularly maternal smoking, is associated with internalizing and externalizing behavioral problems in children [12-14]. Maternal smoking has been linked to mental health issues in children, including symptoms of melancholy, anxiety, and suicidal ideation [15]. Parental smoking is associated with the initiation and regular use of smoking among children and adolescents [16]. The direct effects of smoking on children and adolescents may be attributed to an induced biological vulnerability to the addictive properties of nicotine, whereas the indirect effects may be driven by nicotine-induced behavioral problems in childhood [17].

A systematic review and meta-analysis showed that compared with smoking cessation, continued smoking was associated with increased depression, stress, and poor overall mental health [18]. However, to our knowledge, no studies have examined the association between parental smoking (as reported by parents) and adolescents’ mental health in Asian cultures. Furthermore, as Asian cultures are more receptive to men engaging in smoking than women, societal acceptance of female smoking remains low despite increasing smoking rates in women [19]. In South Korea, the prevalence of depression among adolescents reached 20.3% in 2021, and smoking remains a significant public health concern, with 4.5% of high school students reporting current use [20]. These high rates of mental health issues and smoking highlight the urgent need to understand the family-related factors influencing adolescent well-being in this cultural context.

We expected that parents’ sex and smoking status would have differential effects on the mental health outcomes of adolescents. This study examined the association between parental smoking, with a particular focus on maternal smoking, and adolescent mental health in the South Korean cultural context. The current findings will contribute to a more nuanced understanding of these relationships.

## Methods

### Study Design and Population

This study analyzed data from the Korea National Health and Nutrition Examination Survey (KNHANES), a cross-sectional, nationally representative survey conducted by the Korea Centers for Disease Control and Prevention from 2012 to 2017. The survey used a stratified multistage probability sampling design to draw a sample representative of the entire South Korean population. The KNHANES included health interviews, health behavior surveys, nutrition surveys, and health examinations.

**Figure 1.**
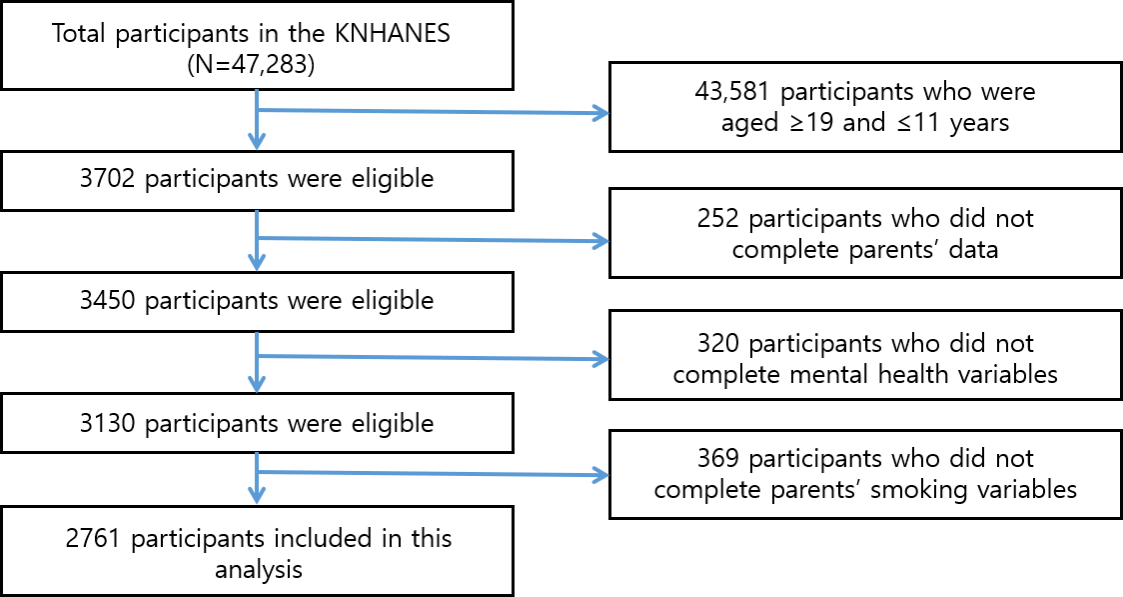
Schematic depicting the study population. KNHANES: Korea National Health and Nutrition Examination Survey.

Of the 47,283 participants enrolled in the KNHANES between 2012 and 2017, we included those aged 12‐18 years (n=3702). Participants with missing parental (n=252), mental health (n=320), or parental smoking (n=369) variables were excluded. Finally, we examined the data of 2761 adolescents enrolled in the KNHANES (Figure 1).

### Research Variables

#### Baseline Characteristics

We recorded the following baseline characteristics of adolescents: age, sex, household and personal income levels, smoking status, and alcohol consumption. In addition, we recorded the following baseline parental characteristics: age, education level, current economic activity, BMI, waist circumference, smoking status, and alcohol consumption.

We classified household and personal income based on equivalent income,

$\frac{average\;monthly\;household\;income}{\sqrt{number\;of\;family\;members}}$,

designating values reflecting the lowest 25% as the first quartile, and the subsequent 3 levels (25% each) as the second, third, and fourth quartiles. We categorized parental education levels as “below university graduation” and “university graduate or higher.” We organized current economic activity as “yes” or “no” to represent whether parents were currently employed or unemployed. We classified smoking status according to whether participants currently smoked. We considered participants who consumed more than 30 mL of alcohol per day as heavy drinkers.

Well-trained examiners performed anthropometric measurements during the study period. We acquired measurements for height and waist circumference to the nearest 0.1 cm using a portable stadiometer (Seca 225, Seca) and a calibrated ruler (Seca 200, Seca) after exhalation and from the narrowest point between the lower borders of the ribcage and the iliac crest. We measured weight to the nearest 0.1 kg using a calibrated balance-beam scale (GL-6000‐20; G-tech). All instruments were calibrated regularly to ensure measurement accuracy, and inter-rater reliability tests were conducted periodically to minimize measurement bias. We calculated BMI as weight divided by height squared (kg/m^2^).

#### Smoking Status

Smoking status was assessed using the question, “Are you currently smoking?” Participants could respond with “I smoke every day,” “I smoke sometimes,” “I smoked in the past, but I don’t smoke currently,” or “not applicable.” We classified participants’ smoking status as “current smoker,” “ex-smoker,” or “never smoked,” with “current smoker” including “every day or occasional smoking.”

#### Mental Health

The mental health variables included cognitive stress, experiences of melancholy, and suicidal ideation. The following questions were developed and reviewed by a panel of experts for epidemiological research, ensuring their validity as single-item questions. For cognitive stress, we classified the responses of “I feel very stressed,” “I feel stressed a lot,” and “I feel a little stressed” as “yes” and the response of “I hardly feel stressed” as “no.” We determined whether participants experienced melancholy based on the question, “Have you felt sad or depressed for at least 2 consecutive weeks within the last year to the extent that it disturbed your daily life?” To this, participants could answer “yes” or “no.” We assessed suicidal ideation using the question, “Have you thought of committing suicide in the last year?” The possible responses included “yes” and “no.”

We did not measure “experiences of melancholy” as a parental mental health variable in this study; however, we measured the difference in adolescents’ mental health based on whether their parents were diagnosed with depression. We defined participants as being diagnosed with depression if they responded “yes” to the question, “Have you ever been diagnosed with depression by a doctor?”

### Statistical Analysis

We used the SAS survey procedure (version 9.3; SAS Institute Inc) to run a complex sample design and analyze the survey data. Mean and SE values were used to represent continuous variables, and mean percentage and SE values were used to represent categorical variables. We used an ANOVA and the chi-square test to compare the baseline characteristics and mental health variables of adolescents and parents. Subsequently, we performed multiple logistic regression analyses to evaluate the association between adolescents’ mental health variables and differences in parental smoking status. We examined the odds ratios (ORs) and 95% CIs after adjusting for age, sex, smoking status, alcohol consumption, and household income. In addition, we examined the ORs and 95% CIs in the association between adolescents’ mental health and mothers’ current smoking status and cognitive stress.

### Ethical Considerations

The original data for the KNHANES were collected after being approved by the institutional review board of the Korea Centers for Disease Control and Prevention. Written informed consent was obtained from all participants prior to the original data collection. As this study is a secondary analysis using publicly available and deidentified data, it was determined to be exempt from a separate institutional review board review (number 1041078-202106-HRSB-172-01). No compensation was provided to participants for this specific analysis.

## Results

### Adolescents’ Mental Health According to Adolescents’ and Parents’ Baseline Characteristics

Table 1 shows adolescents’ mental health according to their baseline characteristics and mental health variables, along with those of their parents.

**Table 1.** Adolescents’ mental health according to adolescents’ and their parents’ baseline characteristics (N=2761).

Variables	Cognitive stress			Experience of melancholy			Suicidal ideation		
	No (n=2059)	Yes (n=702)	*P* value	No (n=2536)	Yes (n=225)	*P* value	No (n=2600)	Yes (n=161)	*P* value
Adolescents									
Age (years), mean (SE)^a^	15.1 (0.05)	15.31 (0.08)	.02	15.12 (0.04)	15.52 (0.14)	.007	15.15 (0.04)	15.1 (0.16)	.73
Sex (male), mean % (SE)^b^	54.28 (1.19)	47.32 (2.10)	.005	53.78 (1.07)	38.69 (3.85)	<.001	53.47 (1.06)	37.24 (4.51)	<.001
Household income (lowest Q1), mean % (SE)	9.27 (0.95)	10.76 (1.40)	.34	9.43 (0.85)	12.03 (2.52)	.27	9.32 (0.81)	14.89 (4.01)	.09
Current smoker (yes), mean % (SE)	13.55 (0.90)	21.70 (1.91)	<.001	14.83 (0.86)	24.31 (3.35)	.001	14.98 (0.85)	25.84 (4.41)	.003
Heavy drinker (yes), mean % (SE)	31.93 (1.28)	41.48 (2.19)	<.001	32.61 (1.18)	53.36 (4.19)	<.001	33.38 (1.17)	49.83 (4.28)	<.001
Mothers									
Age (years), mean (SE)	44.15 (0.13)	44.62 (0.19)	.03	44.25 (0.12)	44.48 (0.35)	.52	44.26 (0.11)	44.41 (0.47)	.75
Education (≥ university), mean % (SE)	39.53 (1.55)	38.81 (2.32)	.77	39.62 (1.47)	36.32 (3.64)	.39	40 (1.44)	29.27 (4.10)	.02
Current economic activity (yes), mean % (SE)	66 (1.47)	63.2 (2.25)	.26	65.79 (1.34)	59.68 (3.99)	.12	65.29 (1.33)	65.15 (4.68)	.98
BMI (kg/m^2^), mean (SE)	23.24 (0.09)	23.54 (0.17)	.10	23.29 (0.09)	23.58 (0.3)	.35	23.3 (0.09)	23.61 (0.38)	.42
Waist circumference (cm), mean (SE)	77.2 (0.25)	77.99 (0.46)	.11	77.36 (0.24)	77.81 (0.76)	.56	77.38 (0.24)	77.66 (0.94)	.77
Current smoker (yes), mean % (SE)	4.19 (0.61)	7.5 (1.24)	.006	4.66 (0.57)	9.16 (2.20)	.009	4.69 (0.56)	10.32 (3.24)	.02
Heavy drinker (yes), mean % (SE)	5.69 (0.71)	6.91 (1.27)	.36	5.71 (0.66)	9.24 (2.42)	.09	5.88 (0.66)	7.93 (2.65)	.39
Cognitive stress (yes), mean % (SE)	25.34 (1.41)	33.95 (2.16)	.001	26.42 (1.22)	39.78 (3.59)	<.001	26.69 (1.18)	40.64 (4.80)	.002
Diagnosis of depression (yes), mean % (SE)	3.89 (0.52)	5.21 (0.93)	.16	4.07 (0.48)	5.94 (1.79)	.23	4.1 (0.48)	6.25 (2.30)	.27
Fathers									
Age (years), mean (SE)	47.01 (0.15)	47.54 (0.23)	.04	47.11 (0.14)	47.41 (0.42)	.49	47.16 (0.14)	46.82 (0.53)	.54
Education (≥ university), mean % (SE)	50.72 (1.89)	45.87 (2.90)	.10	49.87 (1.81)	45.76 (4.69)	.38	49.89 (1.78)	43.87 (5.69)	.29
Current economic activity (yes), mean % (SE)	94.51 (0.88)	95.07 (1.15)	.66	94.67 (0.83)	94.39 (1.76)	.88	94.88 (0.77)	90.93 (4.25)	.24
BMI (kg/m^2^), mean (SE)	24.89 (0.11)	24.70 (0.16)	.28	24.84 (0.10)	24.88 (0.26)	.90	24.84 (0.10)	24.99 (0.35)	.67
Waist circumference (cm), mean (SE)	86.12 (0.28)	86.19 (0.45)	.88	86.13 (0.27)	86.26 (0.65)	.84	86.09 (0.26)	86.85 (0.97)	.45
Current smoker (yes), mean % (SE)	42.95 (1.69)	46.15 (2.79)	.30	43.93 (1.59)	41.35 (4.40)	.58	44.11 (1.53)	37.34 (5.62)	.24
Heavy drinker (yes), mean % (SE)	25.46 (1.42)	28.86 (2.37)	.17	26.16 (1.36)	27.68 (4.01)	.71	26.78 (1.34)	18.17 (3.77)	.046
Cognitive stress (yes), mean % (SE)	29.03 (1.56)	32 (2.48)	.27	29.90 (1.44)	28.03 (3.87)	.65	29.6 (1.43)	32.3 (5.15)	.60
Diagnosis of depression (yes), mean % (SE)	2.07 (0.50)	0.42 (0.30)	.01	1.65 (0.41)	1.92 (1.15)	.81	1.73 (0.42)	0.68 (0.68)	.34

a Mean (SE) values represent continuous variables.

b Mean percentage (SE) values represent categorical variables.

**Table 2.** Adolescents’ mental health and maternal smoking status (N=2761).

Maternal smoking status	Cognitive stress			Experience of melancholy			Suicidal ideation		
	Mean % (SE)	Model 1^a^, OR^b^ (95% CI)	Model 2^c^, OR (95% CI)	Mean % (SE)	Model 1^a^, OR (95% CI)	Model 2^c^, OR (95% CI)	% Mean (SE)	Model 1^a^, OR (95% CI)	Model 2^c^, OR (95% CI)
Never smoked	24.33 (1)	1 (ref^d^)	1 (ref)	7.82 (0.63)	1 (ref)	1 (ref)	5.53 (0.53)	1 (ref)	1 (ref)
Ex-smoker	35.19 (5.17)	1.72 (1.08- 2.75)	1.63 (1.04- 2.59)	10.52 (3.07)	1.45 (0.74- 2.85)	1.32 (0.68- 2.55)	10.23 (3.20)	1.97 (0.93- 4.18)	1.74 (0.80- 3.78)
Current smoker	37.83 (5.19)	1.88 (1.20- 2.95)	1.65 (1.06- 2.56)	15.07 (3.71)	2.09 (1.20- 3.65)	1.64 (0.93- 2.89)	12.39 (3.84)	2.39 (1.17- 4.88)	1.72 (0.88- 3.38)

a Model 1: adjusted for adolescents’ age and sex.

b OR: odds ratio.

c Model 2: adjusted for adolescents’ age, sex, household income, current smoking status, and heavy drinking status.

d ref: reference.

Regarding adolescents’ cognitive stress, we found significant differences according to their age (*P*=.02), sex (*P*=.005), current smoking status (*P*<.001), and heavy drinking status (*P*<.001); maternal age (*P*=.03), current smoking status (*P*=.006), and cognitive stress (*P*=.001); and paternal age (*P*=.04) and diagnosis of depression (*P*=.01).

For adolescents’ experiences of melancholy, we observed significant differences according to their age (*P*=.007), sex (*P*<.001), current smoking status (*P*=.001), and heavy drinking status (*P*<.001), as well as maternal current smoking status (*P*=.009) and cognitive stress (*P*<.001). No significant associations were found for the paternal variables.

Suicidal ideation showed significant differences according to adolescents’ sex (*P*<.001), current smoking status (*P*=.003), and heavy drinking status (*P*<.001); maternal education level (*P*=.02), current smoking status (*P*=.02), and cognitive stress (*P*=.002); and paternal heavy drinking status (*P*=.046).

### Association Between Adolescents’ Mental Health and Parental Smoking Status

Table 2 presents the association between adolescents’ mental health and maternal smoking status. In Model 1 of the logistic regression analysis, we adjusted for adolescent age and sex. In Model 2, we adjusted for adolescent age, sex, smoking status, alcohol consumption, and household income. The analysis revealed that maternal smoking status was significantly associated with adolescents’ cognitive stress and melancholy (*P*=.03). The ORs for adolescents’ cognitive stress were 1.72 (95% CI 1.08‐2.75) and 1.63 (95% CI 1.04‐2.59) in Models 1 and 2, respectively, in the group with mothers who were ex-smokers; the ORs were 1.88 (95% CI 1.20‐2.95) and 1.65 (95% CI 1.06‐2.56) in Models 1 and 2, respectively, in the group with mothers who were current smokers. The OR for adolescents’ experience of melancholy was significant in Model 1 at 2.09 (95% CI 1.20‐3.65) only in the group with mothers who were current smokers. In addition, the OR for adolescents’ suicidal ideation was significant in Model 1 at 2.39 (95% CI 1.17‐4.88) only in the group with mothers who were current smokers. Paternal smoking was not significantly associated with adolescents’ mental health.

### Association Between Adolescents’ Mental Health and Mothers’ Current Smoking Status and Cognitive Stress

**Figure 2.**
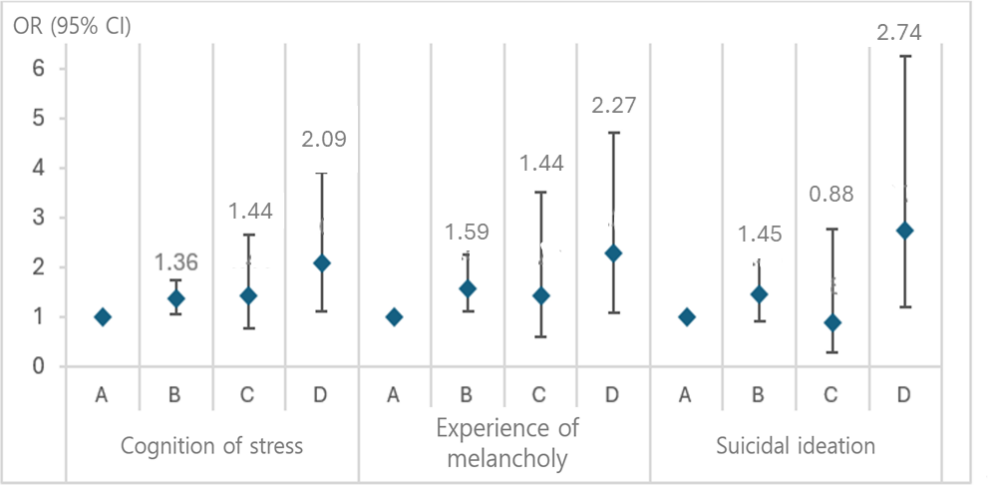
Association between adolescents’ mental health and mothers’ current smoking status and cognitive stress (N=2761). OR: odds ratio.

Figure 2 shows the association between adolescents’ mental health and mothers’ current smoking status and cognitive stress. We classified mothers into 4 groups depending on their current smoking status and cognitive stress: Group A (reference group) for mothers who were “not current smokers and without cognitive stress,” Group B for mothers who were “not current smokers but with cognitive stress,” Group C for mothers who were “current smokers but without cognitive stress,” and Group D for mothers who were “current smokers with cognitive stress.” The significant ORs were 1.36 (95% CI 1.06‐1.75) in Group B and 2.09 (95% CI 1.12‐3.90) in Group D for adolescents’ cognitive stress, and 1.59 (95% CI 1.11‐2.27) in Group B and 2.27 (95% CI 1.10‐4.71) in Group D for adolescents’ experiences of melancholy.

However, adolescents’ suicidal ideation was significant for only Group D (OR 2.74, 95% CI 1.21‐6.23). Figure 2 shows that adolescents’ mental health ORs in Group D (current smokers with cognitive stress) were higher than those in Group A (reference group).

## Discussion

### Principal Findings

This study examined the association between parental smoking status and the mental health of their adolescents in South Korea, specifically focusing on cognitive stress, melancholy, and suicidal ideation. Furthermore, we analyzed these factors separately for mothers and fathers to identify any differences in the effects.

We observed a significant relationship between adolescents’ cognitive stress and their parents’ age and current smoking status. As parents age and their physical aging progresses, their employment stability may decrease; in addition, they often have to care for their own older parents. This can expose their children to various stressful situations [21]. In addition, mental health problems such as parental depression can negatively affect children’s cognitive health. However, strengthening protective factors across various areas of adolescents’ lives may help prevent psychological health problems among adolescents [22]. Therefore, researchers should use multiple approaches to improve parental mental health and reduce perceived stress among adolescents.

Mothers’ current smoking status and cognitive stress influenced their adolescents’ experiences of melancholy, whereas fathers’ smoking status had no such significant effect. Parent–child communication is related to adolescents’ life satisfaction, with their relationships with mothers having a particularly strong influence on girls [23]. In contrast, adolescent aggression and depressive symptoms were associated with increased mother-adolescent conflict [24]. In South Korean culture, the mother-child relationship is notably close [25], which may explain why mothers have a stronger influence on their adolescents’ mental health than fathers. The observed effects of maternal smoking could also be influenced by prevailing social norms. In many Asian cultures, women who smoke may be perceived as violating traditional norms, leading to moral judgments (eg, viewed as less respectable, lacking self-control, or not family-oriented). This social stigma can indirectly affect adolescents’ mental health through social pressure, family reputation concerns, or community gossip.

Reiss et al [26] analyzed data from the German National Health Interview and Examination Survey for children and adolescents, revealing that children of parents with higher education levels had fewer mental health problems in response to stressful life situations than their peers. Moreover, Guerrero et al [27] reported that the children of parents with lower education levels required interventions to address their risk of developing mental health problems due to stressful situations. These results echo those of our study, as parents’ level of education can create high expectations for their children to study or go to school. In addition, the expression of conflict can be attributed to differences in one’s future goals.

Maternal smoking and cognitive stress consistently had significant effects on adolescents’ mental health, as mothers’ smoking status was associated with their adolescents’ cognitive stress, melancholy, and suicidal ideation. Adolescents whose mothers were current smokers and experienced stress exhibited higher cognitive stress, melancholy, and suicidal ideation than those whose mothers did not smoke or experience stress. Amrock and Weitzman [28] analyzed data from the National Health Interview Surveys in the United States to examine the mental health of children aged 4‐17 years and identified a negative effect of parental mental health and sex on adolescent mental health. However, our study found that only maternal cognitive stress significantly affected adolescents’ cognitive stress, melancholy, and suicidal ideation. This could be because in South Korea, mothers are primarily responsible for raising their children; thus, their mental health may have a more significant influence on their children than that of their fathers. Moreover, in a study comparing mothers’ child-rearing stress in the United States and South Korea, South Korean mothers showed substantially higher stress levels than did American mothers [29].

Lee et al [30] found that adolescents’ mental health was significantly associated with maternal mental health and smoking status but not with paternal mental health. This difference may be because fathers often display cooperative and constructive problem-solving behaviors that adolescents tend to emulate. However, their focus on problem-solving may reduce emotional engagement, making interactions feel less supportive than the warmth typically provided by mothers [31]. Moreover, maternal depression can impair mother-child attachment and elevate maternal stress, leading to diminished nurturing behaviors. This creates a stressful home environment, potentially resulting in developmental challenges and emotional difficulties for adolescents [32]. Children may develop psychological symptoms as a result of receiving insufficient support during challenging moments, thereby necessitating the consideration of parental mental health [33]. By addressing parents’ needs through family support programs, health professionals can improve the mental and behavioral health of adolescents as well as the happiness and nurturing nature of parents. Such family support programs can be a part of treatment and prevention [34].

Stress is a significant risk factor for smoking, as individuals often smoke to reduce stress [35]. In South Korea, the negative perception of women smoking likely means that the actual number of female smokers is substantially higher than the officially reported smoking rates [36]. Therefore, women may be more likely to smoke in personal spaces, such as their homes, than in public areas; therefore, children may be more likely to be exposed to their mothers’ smoking.

Our findings provide valuable evidence supporting the influence of parental smoking on adolescents’ mental health. However, this study has some limitations. First, our cross-sectional design limits causal interpretation. We analyzed data from the nationwide KNHANES; therefore, the results are associative rather than causative, restricting our ability to confirm causal relationships between parental smoking and adolescent mental health outcomes. Second, this study focused exclusively on South Korean adolescents and parents; therefore, the results may not apply to populations in different cultural or social environments, and their generalizability may be limited. Finally, the accuracy of self-reported parental smoking status remains a potential issue. The cultural stigma surrounding women engaging in smoking in South Korea may lead to the underreporting of mothers’ smoking behaviors. Future research should consider a wider range of variables and interaction effects to provide a more comprehensive analysis of the factors affecting adolescent mental health and strengthen the understanding of these relationships across cultural contexts. In addition, future studies should incorporate longitudinal data to better understand the causality of these variables.

### Limitations and Recommendations

The cross-sectional design of our study limits the ability to establish causality, while the self-reported nature of data presents a risk of underreporting, especially for maternal smoking due to cultural stigma. We recommend that future research use longitudinal data to better understand the causal relationships between variables. In addition, we suggest incorporating diverse measurement methods and standardized mental health scales to overcome the limitations of single-item, self-reported data.

### Conclusions

We examined the relationship between parental smoking status and the mental health of adolescents in South Korea and found a significant association between adolescents’ mental health and mothers’ current smoking status. Moreover, maternal stress has a substantial association with their adolescents’ well-being. We recommend programs to support mothers in managing their stress without having to rely on smoking and to quit smoking, as this can lead to improvements in their children’s mental health. Therefore, systematic support at the national and domestic levels is required. Future research should explore additional factors affecting both parents’ and adolescents’ mental health.

## Abbreviations

KNHANES: Korea National Health and Nutrition Examination Survey
OR: odds ratio
